# Supplementary material for: Effect of Research Impact on Emerging Camel Husbandry, Welfare and Social-Related Awareness
Source: Animals (Basel). 2020 Apr 30;10(5):780. doi: 10.3390/ani10050780 (PMC7277471; doi:10.3390/ani10050780)
Supplement: Supplementary file 1 [file animals-10-00780-s001.zip › Table S1.docx]

**Table S1.** Results of Shapiro-Francia W’ test with log transformation.

| Variable | Observations (n) | W’ | V’ | Z’ | p-value |
| --- | --- | --- | --- | --- | --- |
| Journal Impact Factor (JCR impact factor) | 1,011 | 0.69648 | 205.275 | 12.215 | 0.00001 |
| Mean JCR Impact | 1,011 | 0.62946 | 250.603 | 12.673 | 0.00001 |
| Total citations of the paper | 1,011 | 0.46087 | 364.623 | 13.533 | 0.00001 |
| Mean number of citations per journal | 1,011 | 0.36028 | 432.270 | 13.922 | 0.00001 |

The values reported under W’ is the Shapiro–Francia test statistics. The test also reports V’, which are more appealing indexes for departure from normality. The median values of V’ are 1 for samples from normal populations. Large values indicate nonnormality. The 95% critical values of V’, which depend on the sample size, are between 1.2 and 2.4 (2.0 and 2.8); see [Royston [1]](#_ENREF_1). There is no more information in V’ than in W’ one is just the transform of the other. Under the Box–Cox transformation, the normal approximation to the sampling distribution of W’, used by Shapiro–Francia test, is valid for 5≤ n ≤1000. Under the log transformation, it is valid for 10 ≤ n ≤5000.

1. Royston, P. Estimating departure from normality. *Stat. Med.* **1991**, *10*, 1283-1293.
