# Supplementary material for: Effect of Research Impact on Emerging Camel Husbandry, Welfare and Social-Related Awareness
Source: Animals (Basel). 2020 Apr 30;10(5):780. doi: 10.3390/ani10050780 (PMC7277471; doi:10.3390/ani10050780)
Supplement: Supplementary file 1 [file animals-10-00780-s001.zip › Table S2.docx]

**Appendix B**

**Table S2.** Spearman's rank-order correlations between impact factor related variables for camel research documents.

| **Spearman’s rho** | JCR impact factor per paper publication year | Mean JCR Impact per journal in the whole period | Total citations of the paper | Mean number of citations per journal |
| --- | --- | --- | --- | --- |
| JCR impact factor per paper publication year | 1.000 | 0.845 | 0.051 | 0.289 |
| Mean JCR Impact per journal in the whole period | 0.845 | 1.000 | 0.145 | 0.358 |
| Total citations of the paper | 0.051 | 0.145 | 1.000 | 0.505 |
| Mean number of citations per journal | 0.289 | 0.358 | 0.505 | 1.000 |
